# Integrating informal providers into a people-centered health systems approach: qualitative evidence from local health systems in rural Nigeria

**DOI:** 10.1186/s12913-016-1780-0

**Published:** 2016-09-29

**Authors:** Maia Sieverding, Naomi Beyeler

**Affiliations:** Global Health Sciences, University of California San Francisco, 550 16th Street, 3rd floor, Box 1224, San Francisco, CA 94158 USA

**Keywords:** People-centered health systems, Informal providers, Medicine vendors, Frontline healthcare workers, Health worker motivation, Referrals, Nigeria, Community health

## Abstract

**Background:**

The presence of a large informal healthcare sector in many low- and middle-income countries poses both challenges and opportunities for achieving a people-centered health system. However, few studies have considered how informal providers may fit into a people-centered health systems approach. We examine the self-described roles and motivations of informal medicine vendors and public healthcare workers in rural Nigeria, as well as interactions between them, with the aim of identifying how local health systems may be reoriented for improved service delivery through a people-centered approach.

**Methods:**

We analyzed data from in-depth interviews with 70 medicine vendors and 21 staff of public health facilities in 30 villages across Kogi, Kwara and Enugu states in Nigeria. Interview guides covered the respondent’s or her facility’s role in providing health services to the local community, motivation to work in her respective profession, and relationships and interactions with other frontline healthcare providers. Data were analyzed in Atlas.ti using an open coding approach.

**Results:**

Both medicine vendors and staff of public health facilities viewed themselves as fulfilling an essential primary healthcare function in their villages, and described their main motivation as the desire to help their communities. Medicine vendors were acknowledged by both groups to play an important role in providing care close to underserved rural communities, but within a limited scope of practice. Vendors described referring cases beyond their self-defined capacity to the local public facility. Health facility staff also sent clients to vendors to purchase drugs that were out of stock. However, referrals were informal and unspecific in nature, and the degree to which relationships between vendors and health facility staff were collaborative was highly context-dependent despite their recognized interdependencies in health services provision.

**Conclusions:**

Policies aimed at fostering people-centered health systems should consider the role of informal providers in the delivery of integrated care. In the context of our rural study sites in Nigeria, supporting stronger and more consistent linkages between medicine vendors and public health facilities is a key step towards improving health service delivery.

## Background

There has been a recent push within health systems practice and research to adopt a people-centered health systems (PCHS) approach [1–4]. The PCHS approach recognizes that health systems are composed of a wide range of human actors, including service users, healthcare providers, regulators and policymakers, whose actions and decisions shape the system’s functioning [1, 3, 4]. PCHS is thus a holistic approach that argues for the orientation of service delivery around the needs of users and their communities. This not only includes locating services close to communities [3], but also emphasizes the integration of health services, i.e. the delivery of health services such that users receive a continuum of care through different “levels and sites of the health system” [2]. This focus on integration of services and continuity of care is motivated in part by the fragmentation of health service delivery in many low- and lower-middle income countries, which is oftentimes exacerbated by the presence of a large cadre of informal health providers that is difficult to regulate [2]. Understanding the motivations and practices of frontline healthcare providers, including informal providers, is therefore central to the design and implementation of PCHS approaches to health systems strengthening.

Informal healthcare providers encompass a wide range of practitioners who provide services for which they do not have formal medical training or that are outside the boundaries of their licensure [5]. Informal providers therefore operate with tenuous legitimacy in relation to the formal health system [6]. Although estimates of the size of the informal healthcare sector vary widely, informal providers are thought to comprise a substantial percentage of all providers and to be the source of a large percentage of care delivered in South Asia and Sub-Saharan Africa in particular [5, 7].

The presence of a large cadre of informal healthcare providers poses three particular challenges for a PCHS approach to service delivery. First, while informal providers may be sought out by care-seekers due to their proximity, ease of access and familiarity to the community [7], the quality of care delivered by informal providers remains a prime concern [7–9]. The lack of integration of informal providers into formal health systems is a particular challenge [5, 10] that prevents proper referrals and contributes to the fragmentation of care that a PCHS approach aims to address. Thus, while informal providers could be key participants in a people-centered health system because of their widespread presence and ability to provide health services close to communities, the utilization of informal providers is also a challenge for quality of service delivery.

Second, through the PCHS lens, frontline health workers are viewed as active participants in shaping the health system. Thus, understanding the factors that shape health worker motivation [11], as well as the roles that they serve within their communities [12], is essential to improving local service delivery. A combination of financial and non-financial rewards are important motivating factors for health workers generally [13, 14], but there is a broad perception that informal providers, as a result of their dual roles as healthcare providers and independent businesses, may be more driven by profit than quality health service provision [5, 6]. However, emerging research indicates that informal providers’ motivations may extend beyond profit maximization to include factors such as social prestige, knowledge acquisition, and community solidarity. For example, medicine vendors in North-West Cameroon have been found to view selling medicines as requiring more knowledge than selling non-health-related commodities, thereby conferring a certain expert status on the seller and a responsibility to be a gatekeeper for access to strong medications [15]. Studies of volunteer community health worker motivation in South Asia and Sub-Saharan Africa have also found social recognition [16–19], moral or religious duty [16, 20], a desire to help the community [18, 19], personal empowerment [17] and acquisition of health knowledge [17, 19] to be important non-financial incentives. An understanding of informal providers’ motivations within a specific context is therefore important for their integration into a PCHS approach.

Thirdly, a central component of a PCHS is continuity of care between different providers to meet the needs of clients [2]. As noted above, informal providers pose a particular challenge in this regard due to weak regulatory structures and their contested relationship with the formal healthcare system in many contexts. For the design of a PCHS, an understanding of the relationships between informal and formal providers operating within the same community, and how these relationships affect communications, referrals, and case management, is needed. However, very few studies have examined how informal providers interact with formal healthcare providers, or how informal and formal providers mutually view their own and each other’s roles in community health service delivery.

In this study, we examine interactions between informal drug vendors, known as Proprietary and Patent Medicine Vendors (PPMVs), and formal public healthcare workers in selected rural communities in Nigeria in order to identify how local health systems may be reoriented for improved service delivery through a PCHS approach. Specifically, we aim to (1) compare the roles of PPMVs and public health facilities in providing services to their rural communities, (2) examine PPMVs’ and public healthcare workers’ motivations for working in their respective professions, and (3) analyze the interactions between these two forms of healthcare provider with respect to service integration and continuity of care.

### The role of PPMVs in the local primary healthcare system in Nigeria

PPMVs are retail drug outlets owned and operated by persons that may not necessarily have formal training in pharmacy; many PPMVs learn the trade through an apprenticeship with a more senior shop owner [21]. PPMVs are a legal category of drug retailer, and were established by the Nigerian Ministry of Health to provide access to medicines in underserved communities [22]. They are permitted to sell a set list of pre-packaged, over-the-counter medicines, but are prohibited from selling prescription medicines such as antibiotics, or conducting invasive procedures, such as administering injections [23]. Although there are concerns about the quality of services provided by PPMVs and the degree to which they comply with scope of practice and licensing regulations [9, 24–26], they provide a major source of care. Nationally, PPMVs are the first source of care for up to 55 % of illness episodes in children under age five [27, 28].

The ubiquitous presence and frequent use of PPMVs in Nigeria can be contextualized within a public health system that is decentralized and faces a number of challenges in delivering services. The most direct responsibility for primary healthcare service delivery in Nigeria lies at the local level [1, 29]. Each ward, a local administrative unit, should have a government-run Primary Health Center (PHC) that provides child and maternal healthcare, nutrition, communicable disease control, non-communicable disease prevention, and health education. There are also public health facilities lower than PHCs, such as dispensaries and health posts, that are intended to serve smaller communities [30], and some communities are served by private health facilities. Although some basic services, such as immunization or antenatal care, are offered by many public facilities for free, user fees vary by service and facility [30, 31]. Public facilities in a number of states have been found to have poor infrastructure [30], be understaffed (particularly of more highly trained personnel such as doctors), and experience high staff absenteeism [29–31].

Given these challenges with the formal healthcare system, PPMVs serve as an important alternative source of care and are widespread in the country. A census of PPMVs across 16 states found that there are an average of 19 shops per 100,000 population in the northern states and 26 shops per 100,000 in the southern states. The comparable figures for public and private health facilities were 18 and 17 per 100,000 population, respectively [32]. PPMVs are a particularly important source of care for poor and rural communities [33, 34], where they play a key role in providing access to medicines. Public health facilities, especially in rural areas, have been found to experience stock outs and lack medicines for basic services [30, 31]. Access to pharmacies in rural areas is also limited; there were 2639 registered retail pharmacies in Nigeria in 2005, compared to an estimated 200,000 PPMVs [22].

## Methods

The data for this study are drawn from in-depth interviews with PPMVs and the staff of public health facilities in rural areas of Kogi and Kwara states in North Central Nigeria and Enugu state in the South East region. Kogi and Kwara were selected due to the relative lack of research on PPMVs from the North Central region, whereas Enugu was chosen as a comparison site because much existing evidence on PPMV services and utilization comes from the South East [9]. The interviews were conducted as part of a larger mixed-methods study on the role of PPMVs in providing child health services [32, 35–37].

### Site and sample selection

We used a community-based sampling approach for the study, focusing on rural areas because these are where PPMVs tend to comprise a larger portion of available health services [32]. Within each study state, we selected one or two Local Government Areas, which typically contained one secondary-level district hospital. In each Local Government Area, we then selected two or three wards that were predominantly rural and had a mix of villages in terms of accessibility to health facilities, main roads, and nearby urban centers. In each ward, we selected between four and eight villages in which to conduct interviews, based primarily on the presence of health facilities or PPMVs within the village. Most villages contained a Primary or Basic Health Center, one or more PPMV shops, and in some cases independently operating private nurses, doctors, and traditional healers. Wards and villages were selected in consultation with local NGOs and community leaders. Data was collected in a total of thirty villages in Kogi (8), Enugu (8), and Kwara (14).

Within each selected village, we attempted to interview the owner of every PPMV shop and one staff member from each public health facility in order to better understand the role of each type of provider. When multiple staff members were present in a health facility, the interview was usually conducted with the most senior staff member. As a result, staff’s positions and qualifications were mixed, although the majority held clinical positions (see Results section for details). In each village, one to nine PPMVs and one to four health facility staff were interviewed, depending on the number of shops and facilities present. Although few PPMVs or health facility staff refused to participate in the study, we were unable to interview some because they were either closed or, in the case of PPMVs, the shop owner was not present. We conducted a total of 70 interviews with PPMVs and 21 with health facility staff across the three states. Of the 70 PPMVs interviewed, 27 were in Kwara, 21 in Kogi and 20 in Enugu. More health facility staff were interviewed in Kwara (11) as compared to Kogi (4) and Enugu (6) due to the greater number of villages covered in Kwara.

### Data collection

Interviews were conducted in August 2013 by local staff trained by the authors using a standard three-day curriculum. A different field team, supervised by the authors, conducted the interviews in each state due to language differences. Interviews were conducted in Yoruba in Kwara, Igbo in Enugu, and either Pidgin English or Igala in Kogi. Permission was sought from the village chief, and Local Government Area- and ward-level leadership of the PPMV professional association to conduct the study in their areas, as well as managing- or district-level public health staff as necessary. Interviewers approached PPMV owners and health staff in their shops or facilities, respectively, explained the study, and obtained verbal informed consent from those interested in participating. Interviews lasted 60 to 100 min for PPMVs and 30–40 min for health facility staff, and were conducted in the respondent’s shop or facility so that she could continue attending to clients as needed. Interviews were substantially longer for the PPMVs than for health facility staff because, as noted above, the data was collected as part of a broader study that focused primarily on PPMVs and thus more topics were covered in these interviews. Respondents were given a small gift valued at 500 Naira (~US$3.13) for their participation.

Semi-structured interview guides were developed during a pretesting process led by the authors in April and June 2013. The guide for PPMVs covered their self-definition of their role in the community, motivation to become a drug retailer, scope of practice, business and health practices, and typical customer interactions. The guide for health facility staff covered the services provided by the facility and the health services available to the community more broadly, and the respondent’s personal motivation for being a health worker. Guides for both PPMVs and health facility staff also included questions about their relationships and interactions with other healthcare providers in the community, including differences between the role and function of PPMVs and public health facilities, perceptions of the other type of provider, and referral practices between PPMVs and public health facilities. The study received ethical approval from the University of California, San Francisco and the National Health Research Ethics Committee of Nigeria.

### Data analysis

Interviews were digitally recorded and then simultaneously translated into English and transcribed by a local team. A random selection of interviews in each language group was back-checked for translation and transcription accuracy. The quotes presented in this article have been edited for comprehension as the English translations were sometimes literal and non-standard. Village names have been anonymized and proper names removed to preserve confidentiality.

We conducted the analysis in Atlas.ti using an open coding approach, in which codes, sub-codes, and code hierarchies were derived from the data. We adopted an iterative approach to developing the codebook; each author coded several transcripts and developed a list of codes, which we then merged and reconciled, refining code definitions and hierarchies. We repeated this process several times until only minor adjustments to the codebook resulted. The analysis presented in this article focuses on the code families for PPMVs’ role and motivation to be a drug retailer, as well as codes related referral practices, which included codes in families about customer interaction, scope of practice, and when and where to refer. Additional codes are drawn from families about health providers’ motivation, their perceptions of and connections to PPMVs, and a code family about differences between PPMVs and health facilities that was standard across the two study populations.

## Results

### Sample characteristics

Of the total 70 PPMV respondents, 68 provided background data related to their education and medical qualifications. Thirty-nine had a secondary education and 18 a tertiary-level education; only six had a primary education. Nineteen also had some form of medical training, primarily as a nurse or Community Health Extension Worker (CHEW). Among the health facility staff, nine were CHEWs, six were nurses, and the others had qualifications including midwife and Community Health Officer (a role that supervises CHEWs). The facilities themselves included Primary (10) and Basic (4) Health Centers, Maternity Centers (2), a Child Health Center (1), and hospitals (2). One interviewee was the local Director of Health Services, who rotated between several facilities, and facility type was missing for one interview.

### The self-described role and motivation of frontline health workers in rural Nigeria

In our rural study sites, public health facility staff and PPMVs were the most common types of frontline health workers. Both public facility staff and PPMVs viewed their role as filling an essential primary care function in communities with limited access to healthcare (both in terms of geography and affordability), but PPMVs were described as providing a much more circumscribed set of services than public facilities. Nevertheless, PPMVs and public health facility staff described very similar motivations for practicing their respective professions, in which they highlighted the desire to serve their communities in particular.

#### Proprietary and patent medicine vendors

PPMVs (or “chemists,” as they were referred to locally) across the three study states consistently described their role in their communities as providing a first line source of healthcare for minor ailments. This first-line provider role included dispensing drugs, and at times offering advice on the illness or follow up care. Minor ailments were generally understood to be common illnesses that did not require a trip to a public facility (frequently referred to locally as the “hospital”), so long as the illness responded to treatment and did not progress to a more serious condition.

*[The] role of chemist in this community, we would call it primary health care…Most of the minor illnesses that are not acute or chronic are coming to [the] chemist, like if I’m feeling headache, fever, malaria, diarrhea. Most people report first to the chemist to purchase their drug. After one or two days using the drug and there is no response, then we asked them to go the nearer hospital for their further care.* – PPMV in Panshak, Kwara

In addition to treating common or minor ailments, PPMVs saw providing “first aid” as part of their role as first-line healthcare providers. This included providing immediate symptomatic relief or wound care for customers who were experiencing an acute illness episode and could not get to the hospital immediately. In many cases, this service was intended to facilitate transfer to a hospital, not as curative treatment.

*[If] the person can’t reach the hospital I will give him small medicine so that he will take so that it will help [him] to go hospital; like you give him the medicine that even help him to get the strength to go [to the] hospital* – PPMV in Agbo, Kogi

*Another thing is first aid, if someone has a wound, if you come to our place we may wrap it with bandage then tell you go to the hospital, but we will make sure the bleeding will stop. –* PPMV in Ozonze, Enugu

Correspondingly, the most common motivation that PPMVs gave for operating their shop was to help their rural communities by providing these first-line health services. The centrality of good health to human life – “*health is wealth*” as a number of respondents said – was seen to differentiate selling medicine from selling non-health-related goods, because selling medicines benefitted the community at the same time that it provided the shop owner with an income.

*… if you don’t have good health you won’t be able to do anything at all, [so] now that I am selling drugs, I feel it’s very important, because I know [that] I am contributing to the society. That is why I have decided to sell medicine.* – PPMV in Akpa, Kogi

The desire to gain knowledge about health, which they could use to benefit themselves and their families as well as their communities, was another reason that some respondents mentioned for entering the PPMV field. There was also a sense among some PPMV respondents that selling medicines was a socially desirable profession; some explained their motivation to sell medicines in terms of this being honest work, a profession that society viewed positively, or work that would be blessed by God.

Although the desire to help the community was described by nearly all PPMV respondents as a primary motivation for being a medicine vendor, many also mentioned secondary reasons that connected this altruistic motive with the need to earn an income. Several respondents said they had been interested in health from a young age, or enjoyed related subjects in school, but had not had the opportunity to pursue formal education as a nurse or doctor. They viewed opening a PPMV shop as a way of working in the health field that had much lower barriers to entry. A small number of respondents also had formal medical training, but were unable to find a position in the formal healthcare system or opened the shop alongside their regular job in order to generate income and apply their knowledge.

*The health of children gives me joy… that is why I started selling drugs and not other things…I want to sell drugs to save those children, and also to get the money for feeding my own children.* – PPMV in Oyinye, Enugu

These respondents viewed work as a PPMV as a means of fulfilling both their altruistic and income-generation motivations, and did not portray these motivations as being in conflict.

#### Public health facility staff

As expected, the staff of public health facilities described their facility’s role as providing a wide range of preventative and curative primary health services, largely comprised of antenatal care, delivery, immunization and family planning. A few facilities also reported to be able to provide laboratory testing and higher-level services including in-patient care. Staff also gave health education talks to their local communities, did mobile outreach, and described part of their role as providing a range of advice and counseling to patients. In this sense, they also saw their role as combating lack of knowledge, fear and misinformation that led to poor health behaviors in their local communities.

*Our basic aim is prevention, we’re primary health care, we’re under preventive, so…we educate them on their life style, what they’ll eat, what they’ll do and how they’ll do it in order to prevent ailment so that it will not come.* – CHEW, Primary Health Center in Ekwebelem, Enugu

Although they described their role in providing health services as much broader than PPMV respondents, health facility staff expressed similar motivations for entering their profession, which centered around the desire to help those in need and to serve communities with limited access to health services. At the same time, like PPMV respondents, some public health facility staff described these motives within the context of needing to make a living.

*We are not only working for monetary aspect of it, but bearing in mind that your work is to save life. Because of people’s condition in this community, I decided to park in, in [the] nurses quarter, so that I will be available at any time they are in need of me, because if all of us [staff] will be scarce… people will be suffering a lot*.– Deputy Director of Nursing, hospital in Mbanusi, Enugu

Several of the health facility staff also described having been interested in health and healthcare from a young age, or mentioned gaining health knowledge as a motivation for entering their field, both in terms of the ability to pass that knowledge to others in order to help them, and in some cases to be able to help their own families.

### Providing care close to communities

Although public health facility staff and PPMVs viewed their role as providing primary healthcare, respondents of both types emphasized the particular importance of PPMVs in terms of the provision of care to underserved rural communities. This was particularly true in Enugu, where a number of PPMVs described their role in relation to the poor availability of other health services in their villages.

*What chemists do in this community is enormous because this community is not one where a hospital is nearby…Our help is that we stay closer, stay close to the poor…Those in the village, you understand? So those that are sick in this community, we are the first people that they come to and we give them something…first aid or treat the illness –* PPMV in Ekwebelem, Enugu

Several PPMV respondents in other states similarly described their role in their villages as important because they were “*close to the community*” or the “*grass root*,” which they used to express a sense of sociocultural as well as geographic proximity to the communities in which they worked.

PPMV respondents also described several ways in which they provided their communities with access to health services that might not have been available otherwise. They emphasized that their shop was often open when the local health facility was not, and were available at night or by phone for emergencies. Health facility staff in a number of villages agreed with this perspective, acknowledging the difficulty of maintaining continuous staffing in some rural areas.

*The importance of chemists in this village is one - if I go home, the chemist person stays till night. The chemist person must live around the shop in the place…. While me, I will not live here, like my [boss] does not live here,….here is not our [staff’s] permanent place and there is no one yet who will agree to stay here permanently…* – CHEW, Primary Health Center in Ozonze, Enugu

PPMV respondents also expressed their value to their communities in terms of their ability to provide more affordable access to medicines, whether because of lower prices or flexible payment methods. Some explicitly contrasted this aspect of PPMV practice with the rigidity of payment schedules at public facilities.

*Some sickness, if they take it to the hospital, it will cost them 1000 [Naira], [but] if they get to us the chemist, we may not collect more than 500 or 400. And some don’t have good money at hand, some will say “please have mercy on me, it is this child’s body that is getting hot, I don’t have more than this 500,” … so you will explain, but in the hospital, there is no mercy.* – PPMV in Ayinde, Kwara

*If you go to the hospital [to buy drugs on] credit now, nobody will sell for you, and if you go to all those health center nobody will sell for you, but we have human sympathy to help ourselves… that is why we stay within our community to help them.* – PPMV in Ndah, Kogi

Health facility staff, while not necessarily approving of clients attending PPMVs purely for financial reasons regardless of their medical condition, similarly recognized that this was an important aspect of PPMVs’ practice in rural settings.

*[Villagers] prefer patent medicine dealers because when treating them they pay when they want. They may go there without having money on their hands and when they get money they pay, and if they can’t get money they will go to farm for that person in place of the money.* – CHEW, Primary Health Center in Ekwebelem, Enugu

This financial aspect supported the common understanding of PPMVs as facilitating access to health services in underserved communities, both in terms of proximity and affordability.

### Interactions between PPMVs and health facilities in providing first-line care

Within the context of our rural study sites, many PPMVs and health facility staff knew each other and described a variety of interactions with one another with respect to their professional roles. These interactions were shaped in particular by the perceived limits to PPMVs’ scope of practice.

#### Limits of PPMV practice

Although they viewed their role as filling an essential gap in access to medicines and basic services in their communities, most PPMV respondents also expressed clear limits to the type of health services they could offer. PPMVs’ understandings of the limits of their scope of practice were influenced both by external regulatory and professional guidelines, and by their self-assessments of their abilities. Many PPMV respondents mentioned the legal limits on the drugs they were allowed to sell and their prohibition from providing certain forms of treatment, such as injections. These limits were enforced (although not necessarily in a consistent manner) by the PPMV professional association as well as regulatory agencies, leading some respondents to limit their practices out of fear of punishment if caught doing things that were not allowed.

*We are bound by law and our association is from the top; we cannot just do things anyhow.… there are some drugs we must not sell…Even when we go to buy drugs, we don’t buy such because if we buy them and go against the law of the association, the association will punish [us].* – PPMV in Iseyemi, Kwara

Most PPMV respondents also described having self-imposed limits on the scope of their practice in the shop. They based these limits in large part on their own level of knowledge and training, and consequently discussed them in relation to the illnesses and medicines that they felt comfortable working with.

*…there are some drugs you may not have any idea about them, you won’t want to sell them. That is why I said [it] is beyond my power, because it is not all the medicines they bring that I know much about, but I know that if I carry it and study it very well if I know the use I will sell it to customers if they come to complain about the sickness.* – PPMV in Ndah, Kogi

*If I take the person’s temperature, I will check the person’s pulse. The things that I get as the pulse and temperature will tell me that the illness is more than I can handle. I will tell the person to go and meet medical doctors that they can handle it.* – PPMV in Onyinye, Enugu

The identification of cases as within or beyond their self-defined capacity was the main means by which PPMV respondents reported setting the boundaries of their actual practice in their shops. Likewise, the comfort and ability criteria were used by a number of PPMV respondents to justify providing services that were outside the legal PPMV scope of practice, such as selling antibiotics, performing injections, or providing more advanced treatment and wound care.

*The difference between me and them [other chemists] is that if [some] one gets injured, I can treat the person but [other chemists] don’t have the liver [boldness] to treat someone with a wound…it depends on how your own apprenticeship was, because some learned from a chemist while the person I learnt from also gives treatment.* – PPMV in Agbo, Kogi

*If there is somebody suffering from an illness that has refused to go, since I have experience in things relating to medicine, I do treat these people.* – PPMV in Ani, Enugu

These respondents viewed these practices as acceptable in their own cases specifically because they had received trained on these services, either formally through nursing or CHEW training, or through an apprenticeship, even though they often recognized that these services were not permitted within their legal scope as a PPMV.

#### Referrals from PPMV to public health facilities for treatment

PPMV respondents indicated that the identification of cases they considered beyond their capacity was also the primary criterion they used to decide whether or not to refer a customer to a more highly trained healthcare provider. In addition to this assessment during an initial encounter, most respondents reported that after dispensing medicines, they would refer any customer who did not improve within two or three days. Upon deciding that someone needed referral, most PPMV respondents stated that their preferred place to send their customers was the local public health facility. While nearly all PPMV respondents reported that they referred, in most cases these referrals were general and non-specific, consisting of a recommendation to seek care at a health facility rather than a concrete referral to a specific health provider. There were, however, some cases in which PPMVs knew local doctors and personally intervened with him or her to ensure a stronger degree of continuity of the customer’s care.

*Last month, someone brought a child with convulsions, and I told [the parents] to take him to the hospital, to Doctor X. But it was raining and [the father] said that there is no way he will find a car to go now. So I called the doctor and asked him what I can give the child. He told me to give paracetamol to cool the fever, and then if the rain stops that they should bring the child.* – PPMV in Ikem Ogu, Enugu

*…the health centre here, most of their staff live in this community, so if someone comes that we cannot help, we might call them on phone and say “Dr. X, come and check him.”* – PPMV in Iyana Share, Kwara

Although none of the health facility staff provided direct examples of referrals from PPMVs in their community, some said that the local PPMVs would refer cases to them in general. Corresponding with how PPMV respondents explained their referral practices, “referral” from the perspective of health facility staff usually appeared to mean that the health provider learned from the patient that she or he had already been to a PPMV for the illness at hand, rather than receiving a message from the PPMV herself. It appeared from both the accounts of health facility staff and PPMV respondents themselves that PPMVs rarely related information about the illness or the drugs dispensed to the health facility, or checked to see whether customers followed up on these referrals.

#### Referrals from public health facilities to PPMVs for drug purchase

Respondents at PPMVs and health facilities reported that customers would also at times be referred back to PPMV shops for drug purchases if the necessary medications were unavailable at the health facility. In some villages, health center staff said that they would also go to the PPMV shop themselves either to fill stock or to purchase drugs for specific patients.

*We work together [with the health center] in that whenever drugs are prescribed to [a customer], they refer [the customer] to come buy [the drugs] from us. Or [the center staff] will come collect the drugs to treat [the customer] and then pay after the treatment.* – PPMV in Shagbe, Kwara

*Some of them [chemists] are professionals….community health [workers] or nurses …you know that people like this, if we refer patients there, they understand what the people will need. Some, even if they don’t have [the drug], if they have a substitute, they can give them because they know that they can be of help through their experience. –* CHEW*,* Basic Health Center in Adegboyega, Kwara

As illustrated by the latter quote, some health facility staff respondents distinguished between PPMVs in their villages based on their medical training, and regarded those with formal training as a better place to refer customers for medicines.

### Village-level variation in the relationships between PPMVs and health facilities

The existence of bidirectional referrals between PPMVs and public health facilities highlights the extent to which their roles in providing primary health services were complementary. In a few instances, respondents themselves described these linkages as such.

*The person brought his child and I told him to go to [the clinic to] be injected. After being injected, [the child] was prescribed drugs to buy…so he was referred to me, because they do refer them to me from the clinic or elsewhere to come and buy drugs.* – PPMV in Olayinka, Kwara

*Immediately after I arrived to this village [the PPMV] came and welcomed me and introduced himself to me as a member of this community and told me the business he does and a member of NAPPMED [PPMV association], that is why I patronize him and I also buy things from him on credit for patients that may not have the money to pay immediately. Then he also refers people to us for cases he cannot handle. –* CHEW*,* Primary Health Center in Akpa, Kogi

However, these linkages in the local health system remained highly informal in nature, dependent upon the strength of individual ties within specific villages. In some villages, particularly in Kwara, facility staff acknowledged that PPMVs were an important access point for primary care, and had some informal referral linkages to PPMVs through clients and drug purchases. A few even said that they would remind local PPMVs to ensure that their drug stocks were approved and not expired.

In other villages, in contrast, health facility staff thought that the presence of PPMVs exacerbated improper care-seeking behaviors among clients, and that PPMVs thus constituted an impediment to the work of the PHC. These respondents expressed concern that PPMVs dispensed inappropriate, ineffective, low-quality or counter-indicated drugs because of their lower levels of training, and because they did not provide diagnostic tests. A number of health facility staff also said that potential clients attended the PPMV when they should have come to the public health facility, and that PPMV shop owners’ customary wait time before initiating referral delayed proper care-seeking at the public facility and caused patients to arrive when their conditions were already severe.

*If [villagers] didn’t patronize the chemist much, then we are supposed to have more patients. But most of [the patients] come when they are serious [very ill]. Initially, they go to buy drugs from the chemist, but when it becomes severe, he will come [to the facility] and tell you that they bought drugs from this chemist man. –* Nursing Officer, Child Health Center in Mbanusi, Enugu

As illustrated by these quotes, some health facility staff’s views of PPMVs contained a degree of professional animosity. A few even viewed PPMVs’ general motivations with suspicion, and thought that PPMVs were willing to try to treat illnesses beyond their level of knowledge.

*Since I came here in April last year, no one has come and said he was referred here by a chemist. In fact, they [chemists] won’t even allow them come, they won’t want to refer them. They will make their own effort whether it is right or wrong.* – Nurse, Primary Health Center in Agbo, Kogi

As a result of these complex dynamics, many health facility staff regarded the PPMVs in their communities as something of a necessary evil: a poor quality source of care, but one that nevertheless was important in providing access to medicines in their underserved communities.

*[There is] no option unless you can travel to [Town X] or [Town Y] to get to a pharmacy, but here it is patent medicine store. You can get [drugs] from them if you are in serious need…They usually don’t go by the law, they will [sell] all these potent drugs…So like there was a time we were looking for ampicillin, we have exhausted our own but they got [it], there was a time we were looking for anti snake venom, they got [it]. They are not supposed to sell it but when you are in need, you don’t query them.* – Nurse, Hospital in Iseyemi, Kwara

## Discussion

A people-centered health system that delivers integrated care close to communities would ideally be achievable through a client-oriented, accessible formal healthcare sector. However, in areas such as the rural Nigerian communities where this study took place, this goal is a long way from being achieved. Given the realities of the situation, care-seekers rely extensively on informal as well as formal providers to meet their different health care needs. In such contexts, we argue that a pragmatic approach to working towards PCHS goals in the shorter-term is to incorporate informal providers, such as PPMVs, that are already located in communities providing basic healthcare services. Building on the motivations of these providers to provide primary care, increase healthcare accessibility, and be integrated into the local healthcare system may help to alleviate some of the access gaps in current provision of care.

The PPMVs interviewed for this study viewed themselves as part of their village health system, not just as businesspeople. Importantly, many local public healthcare workers agreed with this perspective, and acknowledged the particular role of PPMVs in providing care close to communities when and where public facilities were unavailable. Although PPMVs’ and public healthcare workers’ views of each other and the services they provided (or the cost and availability of those services) were not always positive, most recognized a degree of interdependency in their roles providing primary healthcare to underserved rural communities. That these different types of local providers saw themselves as part of a village health system argues for the inclusion of PPMVs in a people-centered approach to improving service delivery in these areas.

While respondents generally agreed that PPMVs played an important role in extending geographic and financial accessibility to care, they understood the PPMV service delivery role as differentiated from, and at a more restricted and lower level of care than, the role of public health facilities. At the same time, there was overlap between the actual qualifications and practices of PPMVs and public healthcare workers. The predominance of CHEWs among the staff of public facilities in the study area, the fact that some staff moonlighted as PPMVs, and that a substantial percentage of PPMVs had qualifications as CHEWS or other trained providers, are all characteristic of the Nigerian healthcare system [9, 30]. As a result, a number of PPMVs were subject to different regulations when operating in their shops (as PPMVs) than outside of their shops (e.g. as CHEWs). The degree to which respondents followed each set of regulations varied from individual to individual. Allowing providers to operate under their training qualifications, regardless of where they sit in the community, might reduce the degree of arbitrariness in compliance with regulations. From a PCHS perspective, this would increase community-level access to services by trained providers, while making it easier to monitor and enforce against poor quality practices by those PPMVs who are overstepping their training.

Both PPMVs and the staff of public healthcare facilities in the study areas explained their motivation to work in the health field in terms of their desire to help their communities while earning a living. That PPMVs expressed non-financial motivations for operating their shops agrees with the literature on volunteer community health worker motivations [16–19], as well as the findings of a study on drug vendors in neighboring Cameroon [15], and supports the broader argument that informal providers – like their counterparts in the formal health system – should not be assumed to be purely profit-oriented. Although PPMVs did not portray their desire to serve their communities as in conflict with their need to generate income, some public health facility staff were suspicious of PPMVs’ true willingness to refer away potential customers, and we are unable to verify the extent to which profit versus altruistic motivations influenced PPMVs’ actual interactions with their customers. Nevertheless, interventions that aim to improve the quality of care provided by PPMVs, including encouraging proper referral practices, should consider the potential to appeal to their non-financial as well as financial motivations for providing quality services. In addition, PPMVs’ desire to be considered part of the healthcare system is in and of itself a non-financial motivation for improved referral and continuity of care. On the other side of the referral relationship, identifying referral points among public healthcare providers who are also located in the community and may have stronger community-oriented motivations is also important.

Another consideration in terms of service integration is the fact that PPMVs’ and healthcare facilities’ roles were differentiated, and largely complementary, in the eyes of many local health systems actors. These complementarities were to some degree realized through the informal referral relationships that existed between PPMVs and public facilities in certain villages. However, these relationships stopped well short of the level of integration that could provide users with continuity of care due to their informal nature and inconsistency across locations, as well as the weak communication accompanying referrals. In a context where PPMVs and public facilities were the two major sources of healthcare, this constitutes a failure of the local health system to provide users with integrated care that can meet their changing needs. From a PCHS perspective, supporting stronger and more consistent linkages between PPMVs and public health facilities is therefore a key step towards improving health service delivery in our rural study sites. Building on existing informal ties between public health center staff and PPMVs through more institutionalized linkages, for example mentoring and training programs or community level meetings, might be a step towards improved continuity and quality of care.

Such measures might also offer a forum for collaboration and addressing professional animosities in localities where existing relationships between the two provider types are weak.

Several limitations are important to consider in the interpretation of our results. We did not employ a random sampling method, so the results cannot be generalized beyond the study area. Interactions between PPMVs and public health facility staff, for example, may be quite different in urban areas. The variability of these interactions even across our study sites furthermore suggest that the dynamics between these two groups are likely to be different in other parts of Nigeria. As the study relied on qualitative methods, we also did not gather data on actual customer referrals either from public facilities or PPMVs, so cannot verify respondents’ claims about their referral practices. There may be some social desirability bias in PPMVs’ responses regarding referral, as they are aware of the legal restrictions on their scope of practice. Similarly, there may be a degree of social desirability bias in both types of providers’ responses about their motivations, and in their presentation of the importance of their role in the local community. However, the use of a purposive sampling method at the village level allowed us to examine the relationships between these two types of providers in greater depth, and given the consistency of responses both within each study population and across the two populations, we believe that respondents were for the most part forthcoming.

## Conclusion

A people-centered health systems approach calls for an in-depth understanding of frontline health workers’ service provision roles and personal motivations, in order to contribute to the design of health services that respond to the needs of users [3, 11, 12]. We argue that informal providers are an important component of this understanding in contexts where they are the source of a significant percentage of health services delivered. The presence of a large cadre of informal providers poses challenges for the PCHS approach [2], but our examination of the roles, motivations and interactions between PPMVs and public health facility staff in rural Nigeria also suggests ways in which this sector might be leveraged for the provision of more people-centered care. The location of informal providers such as PPMVs close to communities, both geographically and socioculturally, is a key opportunity for the PCHS approach, and this study contributes to the growing body of literature indicating that informal providers are motivated to serve their communities. In order to capitalize on this opportunity, however, developing interventions to support consistent collaboration between informal providers and the local formal healthcare system is key to improving both continuity and quality of care.

## Abbreviations

CHEW: Community Health Extension Worker
PCHS: People Centered Health System
PHC: Primary Health Center
PPMV: Proprietary and Patent Medicine Vendor
